# Managing Oroantral Fistula With Chronic Osteomyelitis Using a Nasolabial Flap: A Case Report

**DOI:** 10.7759/cureus.73695

**Published:** 2024-11-14

**Authors:** Gautam Bhambri, Sangita Kalita, Juhimoni Deka, Pronob Paul, Seema Gupta

**Affiliations:** 1 Department of Oral Pathology, Surendera Dental College and Research Institute, Sri Ganganagar, IND; 2 Department of Oral and Maxillofacial Surgery, Maharaja Ganga Singh Dental College and Research Centre, Sri Ganganagar, IND; 3 Department of Oral Medicine and Radiology, Surendera Dental College and Research Institute, Sri Ganganagar, IND; 4 Department of Oral and Maxillofacial Surgery, Kothiwal Dental College and Research Centre, Moradabad, IND; 5 Department of Orthodontics, Kothiwal Dental College and Research Centre, Moradabad, IND

**Keywords:** chronic, nasolabial, oroantral fistula, osteomyelitis, surgical flap

## Abstract

Oroantral fistula (OAF) represents the pathological communication between the oral cavity and maxillary sinus. This condition arises when the structural integrity of the maxillary sinus floor is compromised, resulting in a direct conduit between the sinus and the oral cavity. A less prevalent yet clinically significant contributor to the formation of OAF is chronic osteomyelitis of the maxilla. Therapeutic approaches for OAF are contingent upon the dimensions of the defect, underlying causative factors, and specific patient characteristics. Among the diverse treatment options, nasolabial flap has been recognized as an efficacious and dependable method for the reconstruction of OAF, particularly in cases involving extensive or recurrent defects. The current case report delineates the management of a recurrent OAF in a 52-year-old male patient, which was further complicated by chronic osteomyelitis and managed through nasolabial flap surgical intervention.

## Introduction

Oroantral fistula (OAF) is an abnormal communication between the oral cavity and maxillary sinus, commonly arising as a complication following the extraction of maxillary posterior teeth, trauma, or pathological conditions such as cysts, tumors, and infections [1]. The fistula can result in significant morbidity, including chronic sinus infections, oral sinus reflux of fluids, and difficulty in maintaining oral hygiene. This condition occurs when the integrity of the maxillary sinus floor is compromised, creating a direct pathway between the sinus and oral cavity [2].

One of the less common but significant causes of OAF is chronic osteomyelitis of the maxilla, a persistent inflammatory condition of the bone and marrow caused by bacterial infection [3]. Chronic osteomyelitis weakens the maxillary bone, leading to necrosis and fistulation of adjacent structures, including the maxillary sinus. The disease, often exacerbated by poor immune response or inadequate initial treatment, can lead to large defects in the maxilla that increase the likelihood of OAF [4,5]. Osteomyelitis is more commonly associated with the mandible; however, its occurrence in the maxilla, although rare, poses complex challenges in both infection control and defect closure, particularly when large areas of necrotic bone are involved [6].

The management of OAF requires prompt and definitive treatment to restore the integrity of the maxillary sinus and oral cavity, prevent recurrent infections, and ensure proper functioning [3]. Treatment modalities for OAF vary depending on the size of the defect, the underlying etiology, and patient-specific factors [7,8]. Surgical closure techniques range from local flap procedures, such as buccal advancement flaps and palatal rotation flaps, to more complex procedures involving distant tissue flaps or grafts [2,8]. Among these, the nasolabial flap has emerged as an effective and reliable option for the reconstruction of OAF, particularly in cases of large or recurrent defects [8,9].

The success rate of the nasolabial flap in reconstructing defects such as OAF is generally considered high, with reported success rates ranging from 85% to 100% in various studies [9-11]. This high success rate is attributed to several factors, including the excellent vascularity of the flap, its proximity to the defect site, and the ease with which it can be harvested and rotated into position [10,11]. The nasolabial flap has a robust blood supply from the facial artery, making it resistant to ischemic complications and promoting good healing [8].

The nasolabial flap is a reliable option for reconstructing OAFs, but it can be associated with some complications [7,8]. These include flap necrosis, infection, noticeable scarring along the nasolabial fold, and donor site morbidity such as discomfort or altered sensation [8,10]. There is also a risk of facial asymmetry, fistula recurrence, and flap bulkiness, which may require further intervention [2,12]. Its versatility and reliability make it a valuable tool in cases in which other flaps may be insufficient or fail. The present case report describes the management of a recurrent OAF complicated by chronic osteomyelitis that was treated with nasolabial flap surgery.

## Case presentation

**Figure 1:**
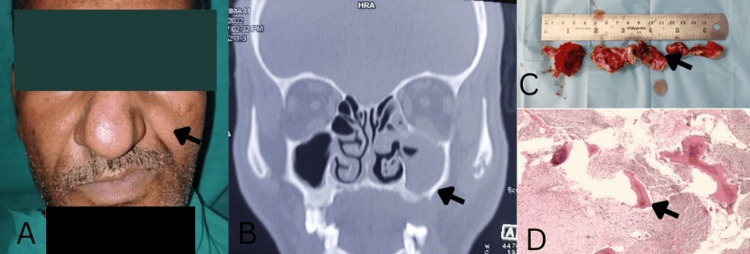
Case description: (A) Extraoral asymmetry with the depressed left side. (B) Cone-beam CT showed an interruption of the floor of the left maxillary sinus. (C) Tissue specimen obtained after surgical exploration. (D) Hematoxylin and eosin-stained section at 40x magnification showed bony trabeculae in a chronic inflammatory background.

A 52-year-old male patient presented to the Department of Oral Surgery in May 2022 at Maharaja Ganga Singh Dental College and Research Centre, Sri Ganganagar, with the chief complaint of communication between his nose and oral cavity, which had been causing regurgitation of food and fluids into his nose while eating and drinking for the past nine months. The patient’s dental history revealed previous extraction of the maxillary left second premolar (25) and left first molar (26), followed by chronic infection in the upper jaw, which was treated with functional endoscopic sinus surgery (FESS) under general anesthesia by an ENT surgeon in May 2021, while the medical history was non-contributory. Upon clinical examination, the extraoral facial asymmetry with a depressed left cheek area (Figure 1A) and intraorally a suspected oroantral fistula in the region of teeth 25 and 26 were detected, with signs of chronic infection, inflammation, and exposure of bone in the maxillary sinus area. Hematological investigations were within normal limits.

A cone-beam computed tomography (CBCT) scan showed bone destruction in the maxilla (Figure 1B), with the formation of an oroantral fistula connecting the oral cavity to the maxillary sinus. Histopathological examination of a biopsy specimen from the lesion (Figure 1C), stained with hematoxylin and eosin, confirmed the diagnosis of chronic osteomyelitis with necrotic bone (Figure 1D). A differential diagnosis of malignancy was considered but ruled out based on histopathology and imaging results. The chronic dental infection with extraction history and formation of an oroantral fistula led to maxillary osteomyelitis. The presence of an OAF can create a direct pathway for bacterial entry from the oral cavity or sinus, leading to infection and potentially contributing to osteomyelitis if not properly treated.

**Figure 2:**
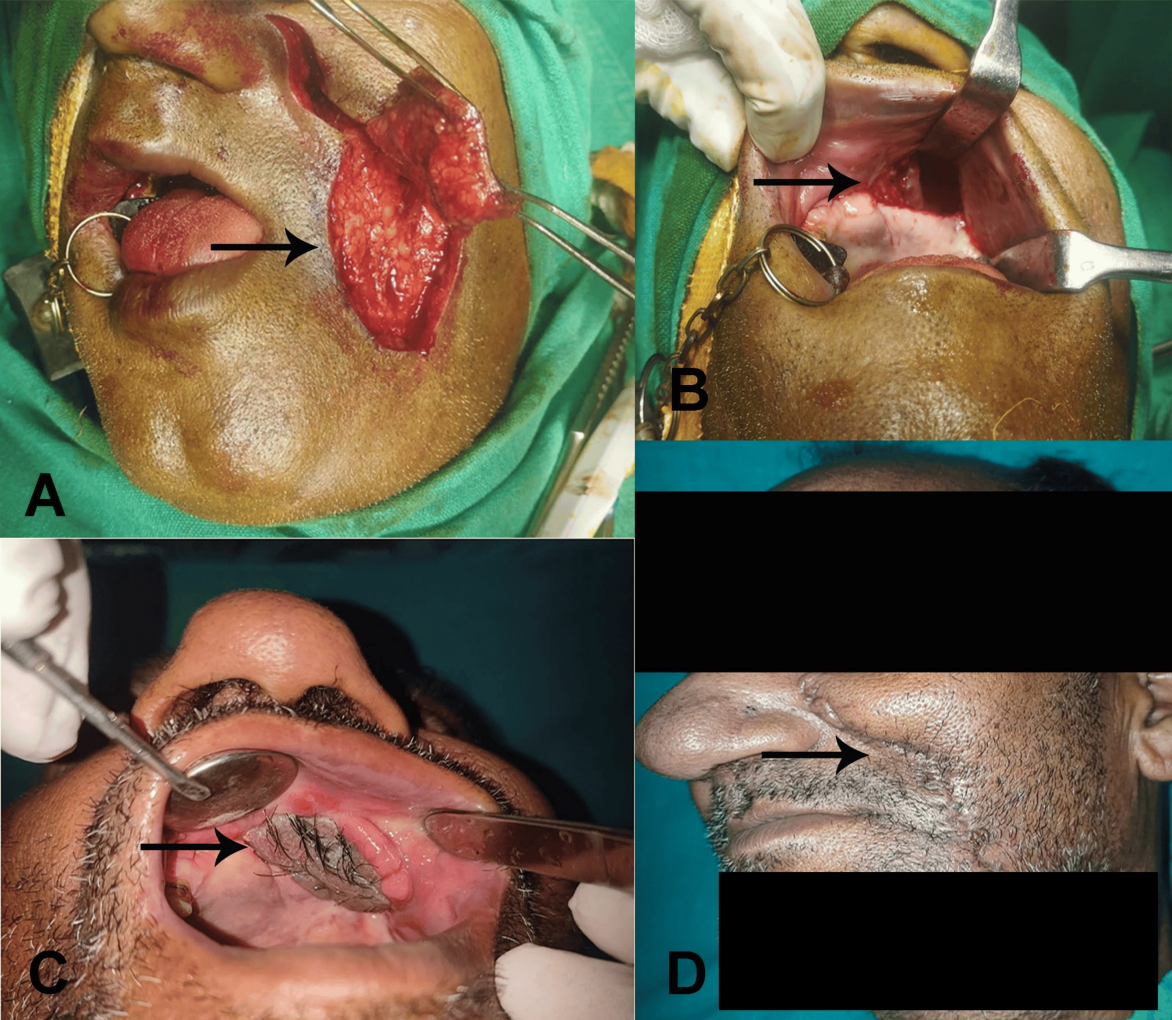
(A) Nasolabial flap. (B) Oroantral fistula. (C) Placement of graft over the fistula. (D) Postoperative healing after two weeks.

The definitive treatment involved nasolabial flap surgery to repair the oroantral fistula caused by chronic osteomyelitis. For large and recurrent cases of oroantral fistulas, the nasolabial flap is often a more suitable choice. The delineation of the flap commenced from the apex of the nasolabial fold extending to the inferior margin of the mandible, succeeded by the surgical incision. The incision traversed the dermal layer and subcutaneous adipose tissue, reaching the level of the underlying musculature. The artery is located in a plane that is situated deep in the facial mimetic musculature and is positioned medially along the nasofacial groove. The flap was elevated in a superior-inferior direction and within the plane of the superficial musculoaponeurotic system, from both terminal points to the area of the central subcutaneous pedicle, which was reliant upon the facial artery. The pedicle was approximately 1 cm lateral to the oral commissure, and its diameter measured approximately 1-1.5 cm (Figure 2A). The flap was transposed intraorally via a small transbuccal tunnel anterior to the pedicle. The transposed flap was employed to encompass the entire extent of the intraoral defect. The suturing was performed utilizing 3-0 Vicryl in the intraoral region. The extraoral defect was subsequently closed in multiple layers employing both 3-0 Vicryl and 4-0 nylon sutures. The surgical procedure was successful, and the fistula was completely closed using a robust nasolabial flap (Figure 2B), ensuring proper sealing and healing (Figure 2C). Postoperatively, the patient was prescribed antibiotics, including amoxicillin with clavulanic acid, to manage the infection, along with pain management medications such as ibuprofen. Oral hygiene measures and a soft diet were recommended to support the healing process.

The healing time for a nasolabial flap surgery typically ranges from three to four weeks (Figure 2D). The pedicle was detached from its origin after the nasolabial flap take-up, which was done to complete the transfer and ensure optimal functional and aesthetic results. The detachment process occurred once the flap had established adequate vascularity in its new position, three weeks post surgery. Detaching the pedicle allowed the flap to integrate seamlessly with the surrounding tissues, minimizing bulk and improving cosmetic outcomes. During this period, the flap established a reliable blood supply and integrated with the surrounding tissues. The patient responded well to the surgical and pharmacological treatment, with no signs of recurrence or complications during the follow-up period. In the initial postoperative phase, the viability of the flap was assessed utilizing the pinprick test, which indicated exceptional vascularization. Intraoral hair growth was frequently noted, which was alleviated through hair plucking under topical anesthesia during follow-up appointments. There was a progressive decline in hair growth, accompanied by subsequent mucosalization of the flap over a duration of six months to one year following surgery. Postoperatively, the healing of wounds at both donor and recipient sites was deemed satisfactory. The bulkiness of the flap diminished over a period of two months, with no notable discomfort experienced by the patient.

## Discussion

OAF recurrence is a challenging clinical issue, particularly in patients with chronic maxillary osteomyelitis. OAF often arises due to a breach in the oral-antral barrier, commonly following dental extractions or infection, leading to pathological communication between the oral cavity and maxillary sinus [2,3]. As seen in the present case, OAF is formed following extraction of the maxillary left premolar and molar teeth (25 and 26). Chronic osteomyelitis significantly complicates the prognosis due to persistent infection, necrosis of bone, and compromised healing in the region, which impairs fistula closure. The presence of chronic infection, as seen in osteomyelitis, can lead to the failure of traditional surgical techniques, such as buccal flap closure, as these procedures rely heavily on the quality of surrounding healthy tissues for successful healing [3,4]. The previous surgical treatment of functional endoscopic sinus surgery failed in our case because of a super-aided chronic osteomyelitis infection.

The nasolabial flap provides a robust alternative for treating recurrent oroantral fistulas, especially in chronic osteomyelitis. This flap offers several advantages over other closure techniques, including buccal flap. One of the primary reasons for its superiority is the rich vascular supply from the facial artery, which enhances healing in infected and compromised areas [7,11]. This characteristic is particularly important in cases of chronic osteomyelitis, where the blood supply to the affected area is often compromised due to necrosis and scarring of the surrounding tissues [4,6]. The present case was treated with a nasolabial flap based on postsurgical advantages. The vascularity of the nasolabial flap promotes rapid healing and provides a better environment for infection control, minimizing the risk of re-infection and fistula recurrence [12]. For cases of OAF with osteomyelitis, where a nasolabial flap may not be the first choice due to its shallow learning curve, several other flap options can be considered as alternatives such as buccal advancement flap, buccal fat pad flap, palatal rotational flap, temporalis muscle flap, and free tissue transfer flap.

In addition to its vascular advantages, the nasolabial flap offers thick, durable tissue that is well-suited for closing larger or recurrent OAFs. The buccal flap, which is commonly used for small fistulas, can fail in cases of recurrent OAF due to its relative thinness and limited reach, which may result in tension at the suture lines and subsequent dehiscence [13]. In contrast, the nasolabial flap provides tension-free closure, which is crucial for the long-term success of fistula repair, especially when dealing with large defects or repeated surgical interventions [11]. The donor site for the nasolabial flap is also concealed within the nasolabial fold, as in our case, ensuring minimal cosmetic concerns post surgery. For recurrent OAFs, where prior closure techniques have failed, their ability to be harvested with minimal tension further reduces the risk of wound dehiscence, making them an excellent option for cases where previous surgeries have led to scarring or fibrosis [14]. Our case did not show any scarring or fibrosis of the tissue, as it was delayed for further consolation after treatment failure.

In the context of chronic osteomyelitis, the nasolabial flap stands out because of its ability to bring healthy, vascularized tissue into an area of poor healing, thus promoting the resolution of chronic infection while simultaneously closing the defect. Studies have demonstrated high success rates of nasolabial flap in OAF closure, even in challenging clinical scenarios involving infection and recurrent fistulas [15]. For instance, Elliott emphasized the importance of the flap's robust blood supply in treating fistulas associated with chronic infections, noting a significantly lower recurrence rate compared with other techniques [12]. Finally, Parvini et al. found the nasolabial flap to be highly effective in managing OAFs in patients with compromised local tissue health due to chronic osteomyelitis, further supporting its role in treating recurrent fistulas [8].

## Conclusions

In conclusion, the nasolabial flap is an excellent surgical option for the closure of recurrent OAFs, particularly in patients with chronic osteomyelitis. The utilization of the nasolabial flap in the realm of facial reconstruction presents a multitude of significant advantages, which encompass not only a remarkable ability to achieve an impeccable match in both color and texture with the specific characteristics of the defect site, but also a highly reliable and robust blood supply that ensures optimal healing and integration, alongside its advantageous anatomical proximity to the defect site that facilitates surgical manipulation and positioning, while concurrently preserving the overall facial countenance and aesthetics, demonstrating minimal morbidity at the donor site, thereby enhancing patient outcomes, and exhibiting an extraordinary level of versatility that allows for its application in various reconstructive scenarios, ultimately culminating in excellent cosmetic results that significantly improved patient quality of life and satisfaction with surgical intervention. The nasolabial flap offers a reliable and durable solution for patients with recurrent fistulas or compromised local tissue health.
